# Circadian Clock Genes Are Essential for Normal Adult Neurogenesis, Differentiation, and Fate Determination

**DOI:** 10.1371/journal.pone.0139655

**Published:** 2015-10-06

**Authors:** Astha Malik, Roman V. Kondratov, Roudabeh J. Jamasbi, Michael E. Geusz

**Affiliations:** 1 Department of Biology, Bowling Green State University, Bowling Green, Ohio, United States of America; 2 Department of Biological, Geological, and Environmental Sciences, Cleveland State University, Cleveland, Ohio, United States of America; 3 Department of Public and Allied Health, Bowling Green State University, Bowling Green, Ohio, United States of America; University College London, UNITED KINGDOM

## Abstract

Adult neurogenesis creates new neurons and glia from stem cells in the human brain throughout life. It is best understood in the dentate gyrus (DG) of the hippocampus and the subventricular zone (SVZ). Circadian rhythms have been identified in the hippocampus, but the role of any endogenous circadian oscillator cells in hippocampal neurogenesis and their importance in learning or memory remains unclear. Any study of stem cell regulation by intrinsic circadian timing within the DG is complicated by modulation from circadian clocks elsewhere in the brain. To examine circadian oscillators in greater isolation, neurosphere cultures were prepared from the DG of two knockout mouse lines that lack a functional circadian clock and from *mPer1*::*luc* mice to identify circadian oscillations in gene expression. Circadian *mPer1* gene activity rhythms were recorded in neurospheres maintained in a culture medium that induces neurogenesis but not in one that maintains the stem cell state. Although the differentiating neural stem progenitor cells of spheres were rhythmic, evidence of any mature neurons was extremely sparse. The circadian timing signal originated in undifferentiated cells within the neurosphere. This conclusion was supported by immunocytochemistry for mPER1 protein that was localized to the inner, more stem cell-like neurosphere core. To test for effects of the circadian clock on neurogenesis, media conditions were altered to induce neurospheres from BMAL1 knockout mice to differentiate. These cultures displayed unusually high differentiation into glia rather than neurons according to GFAP and NeuN expression, respectively, and very few BetaIII tubulin-positive, immature neurons were observed. The knockout neurospheres also displayed areas visibly devoid of cells and had overall higher cell death. Neurospheres from arrhythmic mice lacking two other core clock genes, Cry1 and Cry2, showed significantly reduced growth and increased astrocyte proliferation during differentiation, but they generated normal percentages of neuronal cells. Neuronal fate commitment therefore appears to be controlled through a non-clock function of BMAL1. This study provides insight into how cell autonomous circadian clocks and clock genes regulate adult neural stem cells with implications for treating neurodegenerative disorders and impaired brain functions by manipulating neurogenesis.

## Introduction

Recent studies suggest that cellular circadian clocks may regulate adult neurogenesis and survival of newly formed neurons [1, 2], although circadian studies of neurogenesis in vitro are lacking. During adult neurogenesis, multipotent neural stem cells self-renew and differentiate to generate neurons. The dentate gyrus (DG) and the subventricular zone (SVZ) are two well-understood areas of the mammalian brain containing neural stem cells (NSCs), which are maintained in a unique cellular environment. This niche for NSCs is emulated in vitro within neurospheres that are cultures derived from the DG and SVZ.

Circadian rhythms are endogenous, near-24-hour oscillations in gene expression, physiology, or behavior that are generated in animal cells by two interacting transcriptional-translational feedback loops in which core clock genes (e.g., *Period*, *Cryptochrome*, and *Bmal1*) are rhythmically activated [3]. The circadian clock can couple with the cell cycle [4] and modulate cell proliferation [5]. The circadian oscillator gates the G2/M checkpoint of the cell cycle via clock gene *wee1* [6] and the G1/S transition via clock-controlled genes *p20* and *p21* [4, 7]. Cell cycle control over the circadian clock has also been shown, but is less well understood than cell cycle regulation by the clock [8, 9].

Modulation of neurogenesis and NSC proliferation by an endogenous clock in the DG remains largely unexplored. Cortisol, melatonin, and various neurotransmitters under circadian clock control appear to regulate daily neurogenesis in the central nervous system [10–13]. Circadian rhythms in *mPer2* expression have been reported in hippocampal explant cultures [14], although a separate study did not detect rhythms in the DG in vivo [15]. Hippocampal neural progenitor cells of mice divide more often at night [1, 16]. Disturbed sleep or alterations of circadian clock phase have also been shown to suppress neurogenesis as indicated by reduced expression of doublecortin (DCX), a marker of immature neurons [17].

Circadian rhythms influence learning, cognitive performance, and memory formation across different species [18–20]. Studies describe disruption of circadian rhythms altering learning and memory performance, spatial learning, intra and intersession habituation, place learning, long-term potentiation, and trace fear memory [14, 21–24]. Cryptochrome genes are also necessary for time-place learning [22]. These studies provide much evidence that a functional circadian clock is required for optimal memory formation and persistence [25].

During adult neurogenesis, newly made granule cells produced within the DG form functional hippocampal synapses that appear to provide improved performance of spatial memory tasks, enhanced mood, and neural repair [26, 27]. Because increased neurogenesis is associated with improved cognitive abilities in rodents, optimal circadian control of cell division that introduces new neurons into the hippocampal circuitry may also increase performance. For example, higher levels of cell proliferation in the DG of knockout mice lacking BMAL1 were shown in one study [1], whereas another study described normal proliferation in the DG of *Bmal1* ^*-/-*^ knockout mice [2]. Knockout of BMAL1 using lentivirus shRNA in primary mouse neuronal cultures caused increased cell death, and siRNA-mediated knockdown of *Bmal1* showed similar effects [28]. Overexpression of *Bmal1* in NIH3T3 cells produced an increase in cell proliferation [29]. In contrast, loss of mPER2 functioning increased DG NSPC proliferation [15]

Circadian rhythms in clock gene expression are typically absent in embryonic or multi-potent somatic stem cells but do appear in progenitor cells and more differentiated tissues [30, 31]. One important question is whether adult neural stem progenitor cells (NSPCs) are circadian clock cells that are capable of endogenous, sustained circadian rhythms. Our study identifies circadian rhythms in DG neurosphere cultures independent of rhythmic influences and timing cues from the animal or its environment. We also describe properties of neurosphere cultures from the DG of *Bmal1* ^*-/-*^ and *Cry 1* ^*-/-*^, *2* ^*-/-*^ double knockout mice that lack circadian rhythms. Our results indicate that these circadian clock genes are not required for neurosphere formation in vitro, but their absence slows neurosphere growth, suppresses neuronal fate commitment, and increases apoptosis.

## Materials and Methods

### Animals

Transgenic *mPer1*::*luc* mice [32] were bred and maintained in cycles of 12 h light and 12 h dark to entrain their circadian system. Animal procedures were approved by the BGSU Institutional Animal Care and Use Committee and met National Institutes of Health guidelines. All animal studies using *Bmal1* ^*-/-*^ [33] and *Cry 1* ^*-/-*^, *2* ^*-/-*^ mice [34] were conducted in compliance with the CSU Committee of Animal Care and Use. Animals were 5–8 months old at the time of tissue harvesting, except where noted. *Bmal1* ^*+/-*^ and wild-type (WT) C57BL/6 littermate animals served as controls for the effects of *Bmal1* ^*-/-*^ knockout. *Cry1* ^*-/-*^ and WT littermate mice served as controls to study the effects of *cryptochrome* gene double knockout on sphere growth. When examining fate determination in NSPCs, control neurospheres were prepared from age-matched (6-month-old) WT mice that were *Bmal1* ^*-/-*^ littermates. Neurospheres were also made from 12-month-old *mPer1*::*luc* mice to serve as controls for possible aging effects in *Bmal1* ^*-/-*^ knockouts.

### Neurosphere cultures

Adult male C57BL/6 mice (5–8 months old), *Bmal1* ^*-/-*^ (6–8 months old), *Cry 1* ^*-/-*^, *2* ^*-/-*^ (6–8 months old), and *Cry1* ^*-/-*^ (6–8 months old) animals were euthanized using isoflurane or in a CO_2_ chamber. The genetic background of all knockout mice was C57BL/6. Brains were removed quickly, coronal slices were made with a Brain Blocker (PA 001 Rat; Kopf), and DG and SVZ regions were dissected. The tissue was washed 4–5 times in HBSS and then enzymatically digested with papain and DNAse I (Worthington) for 30 minutes at 37°C, followed by 2–3 washes in DMEM with no added growth factors. The tissue was then mechanically triturated and passed through a 40 μm cell sieve (Falcon; BD Biosciences Discovery Labware, Bedford, MA). The cell suspension was washed and centrifuged 4 times for 5–6 min. The supernatant was discarded and the pellet was re-suspended in stem cell medium (SCM) which consisted of DMEM with 10 ng/ml bFGF, 20 ng/ml EGF (Invitrogen) and 100 U/ml penicillin and 100 μg/ml streptomycin (P/S). Cells were plated at a density of 2.5–3.0 x 10^4^ cells/ml in SCM. Neurospheres were observed after 7–8 days in SVZ cultures and after 10–12 days in DG cultures. These neurospheres were then mechanically triturated and plated in 35-mm dishes. Secondary neurospheres that formed were used for all experiments [35].

### Neurosphere bioluminescence imaging

Culture dishes containing neurospheres in DMEM and 10% FBS (SM) or stem cell medium (SCM) were covered with a temperature-controlled optical window sealed with silicone grease and maintained at 37°C (Cell MicroControls, Norfolk, VA). Spheres were treated for 2hrs with 20 μM forskolin immediately before adding luciferin and were imaged with a back-thinned, back-illuminated CCD camera cooled to -90°C (CH360, Photometrics, Tucson, AZ) and a 50-mm Nikkor f/1.2 lens (Nikon, Melville, NY). Cell dispersals were illuminated by red LEDs when focusing the camera and handling the cultures. Luminescence images were captured with 2 x 2 binning and sequential 1-hr exposures over several days for a maximum of 4 days. Images were analyzed using V++ (Photometrics) and ImageJ (NIH) software.

### Immunocytochemistry

Neurospheres were placed into poly-D-lysine-coated glass-bottom dishes (Mattek) and allowed to attach for 6 hours while in a thin film of SCM, DMEM with 10% fetal bovine serum (FBS) and P/S (SM), or DMEM with B27M (Life Technologies, Grand Island, NY, USA) and P/S (B27M). After the neurospheres attached, 2 ml of medium (SCM, B27M or SM) was added to prevent loss of neurospheres. Neurospheres were fixed in 100% methanol for 10 minutes and standard immunocytochemistry was performed. Immunofluorescence staining was used to identify neural stem progenitor cells, neural progenitor cells, neurons, and astrocytes. Primary antibodies were used at the following dilutions: chicken anti-NeuN (Aves Labs, Tigard, OR, USA) 1:1000; chicken anti-Nestin (Aves Labs) 1:1000; rabbit anti-BetaIII-tubulin (Cell Signaling Technology, Danvers, MA, USA) 1:1000; rabbit anti-Musashi1 (Msi1, Cell Signaling Technology) 1:1000; rabbit anti-GFAP (Cell Signaling Technology) 1:1500; rabbit anti-SOX2 (Life Technologies) 1:500; cleaved caspase–3 (Cell Signaling Technology) 1:500. Samples were rinsed after overnight incubation at 4°C, and were incubated for 2 hours with appropriate Alexa488 and 458-conjugated secondary antibody (Life Technologies). Confocal microscopy of spheres was performed as mentioned in our previous study [35].

### Live/Dead stain

Propidium iodide (PI) is only taken up by cells whose cell membrane integrity is compromised [36]. Neurospheres from both WT and *Bmal1* ^*-/-*^ knockout animals were stained and incubated in PBS with PI (0.02mg/ml) for 5 minutes. PI was then washed out using PBST (0.1% Triton in PBS), and neurospheres were fixed using 100% methanol for 10 minutes. After fixation, neurospheres were washed with PBS to remove excess methanol, and cell nuclei were stained using Hoechst3342 (5 ng/ml in PBS) for 5 minutes. This protocol was modified from a previously published study [37].

### Data analysis

Bioluminescence images were processed and peaks were identified by a method similar to that described in our previous study [35]. Using the peak phase of each circadian cycle, Rayleigh’s test for uniformity was performed using Oriana circular statistics (Kovach Computing Services) to determine whether the phases of circadian rhythms were significantly clustered. The percentage of neural stem cells, neurons, astrocytes, and cleaved-caspase–3^+^ cells was measured using the Metamorph Multi-Wavelength Cell Scoring routine to create segmentation windows that show estimated areas occupied by positively-stained cells. Background intensity was subtracted based on the average intensity measurement from controls in which primary antibody was omitted. Threshold for detection was 30% of the maximum pixel intensity. Overall staining intensity for cleaved-caspase–3 was measured by drawing a region of interest (ROI) around the neurospheres and plotting a histogram to find the mean staining intensity. The means were then compared by using one-way analysis of variance (ANOVA) and T-test.

To evaluate neurosphere proliferation, primary neurospheres were triturated and plated at a density of 3.5–4.5 x 10^4^ cells/ml in a 60-mm tissue culture dish. Neurosphere numbers were counted after secondary spheres were generated in the culture. Medium exchanges were done every 2–3 days and brightfield images were taken on day 14 and day 35 after plating. ImageJ software (NIH) was used to draw an ROI around neurospheres, and size measurements were made at day 14 and 35. ANOVA followed by Scheffe’s post-hoc test was used to compare average neurosphere area between *Cry 1* ^*-/-*^, *2* ^*-/-*^, *Cry1* ^*-/-*^, and WT.

## Results

### Circadian rhythms appear when neurospheres are allowed to differentiate

**Fig 1:**
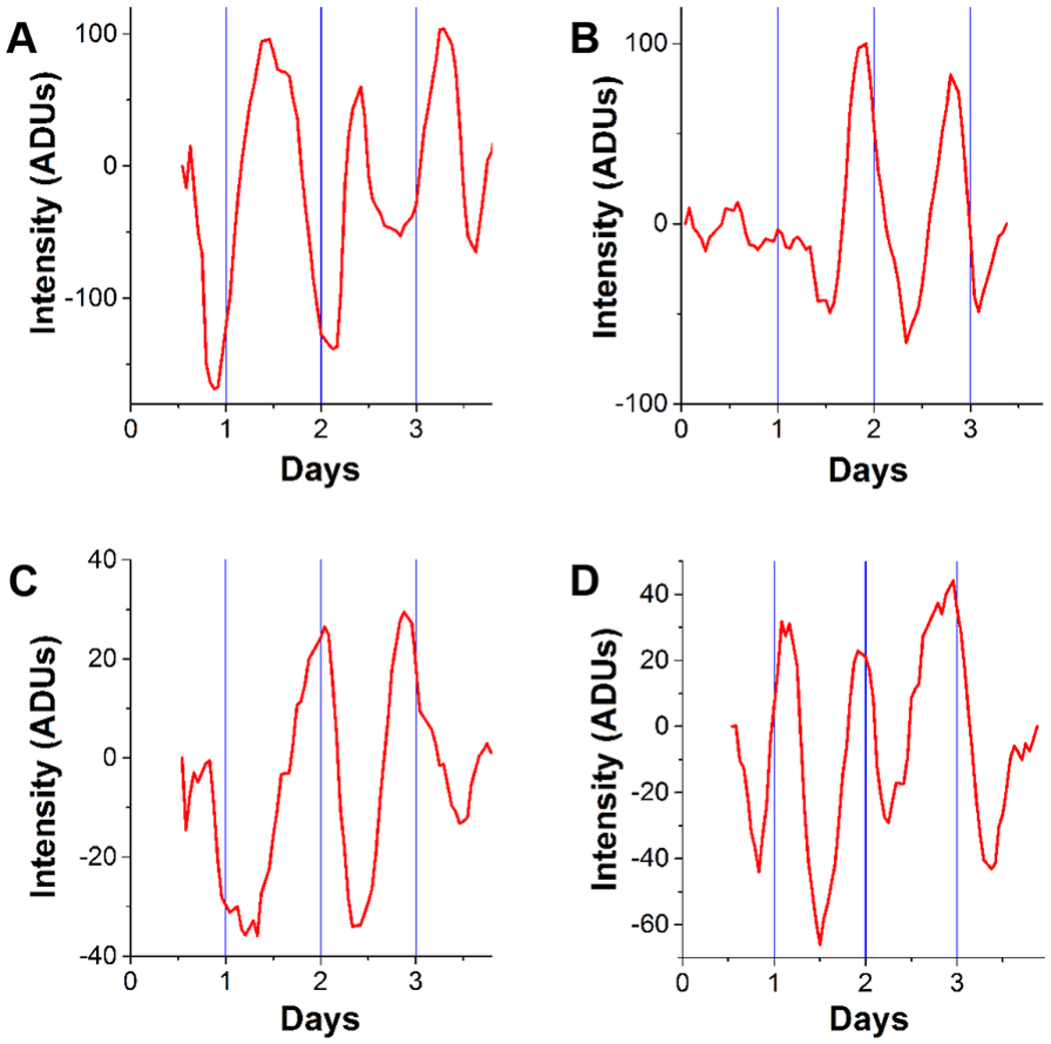
Neurospheres display circadian rhythms in *mPer1* gene expression during early differentiation. DG neurospheres grown in stem cell medium were transferred to serum medium (SM) immediately after a synchronizing forskolin treatment ending at time zero. Shown are 5-point running averages of intensity measurements from images collected hourly. Note that rhythms can be observed as early as the second day in SM. ADUs: analog-to-digital units of the camera.

To identify the status of circadian rhythms in NSPCs, DG neurospheres were prepared from *mPer1*::*luc* mice and imaged in SCM or SM for 4 days after forskolin synchronization. Total bioluminescence intensity recorded over time from each sphere was characterized as either circadian (19 through 29-hr period) or non-rhythmic defined as less than 19-hr period (ultradian), greater than 29-hr period or no significant oscillation [35]. All neurospheres that were imaged while in SCM lacked circadian rhythms (n = 8). One neurosphere in SCM exhibited an ultradian oscillation. Neurospheres were mostly rhythmic in SM (7 of 8 imaged), but one had a low-frequency (18.68 hr) oscillation in *mPer1* gene expression (Fig 1). Significantly more circadian rhythms were recorded in SM than in SCM after forskolin synchronization (Tukey Multiple Comparision post hoc test χ^2^_0.05, 5_ = 11.07, p <0.05, q_∞ 0.05,5_ = 2.472, p <0.05).

Average period of circadian spheres in the SM group, based on peak-to-peak intervals, was 22.12 hrs ±2.64. According to the Rayleigh test the 1^st^ peaks of rhythms recorded in the SM group were significantly clustered (Z = 3.26, p = 0.032). The mean vector occurred at 01:02 ±3.20 (SD) hrs, which was approximately 24 hours after the forskolin treatment. Time 0:00 indicates the end of the 2-hr forskolin pulse.

**Fig 2:**
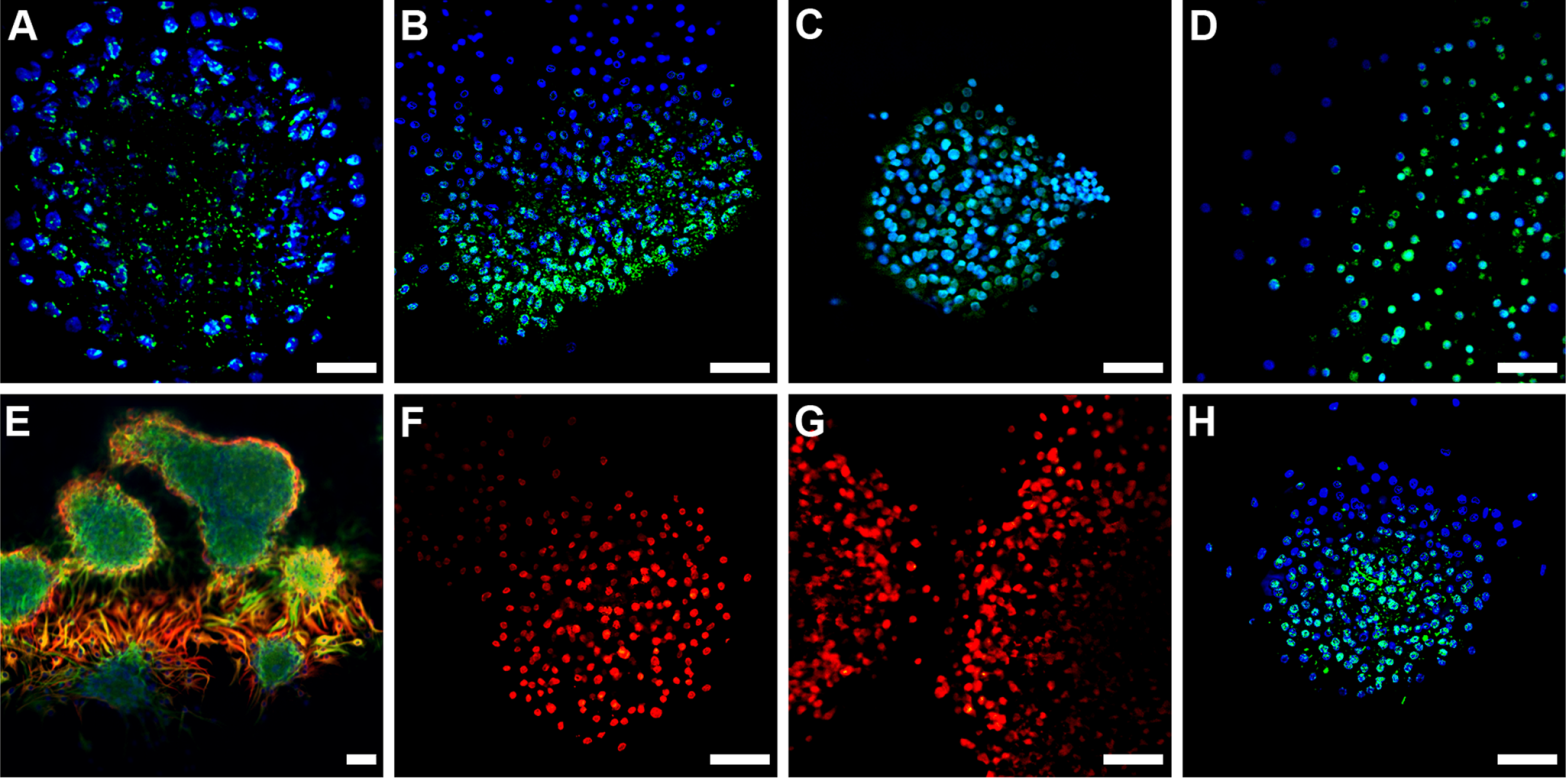
NSPCs are the dominant cell types during the first four days of DG neurosphere differentiation. SOX2^+^ (green) cells after **A:** 1 day in SM, **B:** 4 days in SM. Msi1^+^ (green) cells after **C:** 1 day in SM and **D:** 4 days in SM. **E:** Nestin^+^/GFAP^+^(yellow) cells at day 4 in SM indicating that radial glial-like cells persist after 4 days of differentiation in SM. Also shown are Nestin^+^/GFAP^-^ (green) cells and very few Nestin^-^/GFAP^+^ (red) cells. Neurospheres lack immature and mature neuronal cells after differentiating 4 days in SM as shown by **F:** BetaIII^+^ (green) and **G:** NeuN^+^ (green). Nuclei were stained with propidium iodide (red). **H:** The source of the bioluminescence signal indicated by mPER1^+^ (green) cells in the neurosphere core after 3 days in SM. All nuclei were stained with Hoechst3342 (blue) unless specified. Scale bar = 50 μm.

NSPCs in the DG self-renew and produce neurons and glial cells through a sequence of differentiation stages while identifiable cell markers appear transiently throughout neurogenesis [27]. Circadian rhythms in DG neurosphere cultures were evident by BLI as early as day 1 of differentiation in SM during up to 4 days of imaging. NSPCs were predominant in neurosphere cultures during circadian rhythm ontogeny (Table 1) and were identified by confocal immunofluorescence microscopy using anti-SOX2 (Fig 2A and 2B), anti-Msi1 (Fig 2C and 2D) and anti Nestin and GFAP (Fig 2E) colocalization. BetaIII-tubulin^+^ (immature; Fig 2F) and NeuN^+^ (mature; Fig 2G) neurons were nearly absent after 4 days of differentiation in SM. Immunostaining also detected high mPER1 expression in the neurosphere core (Fig 2H), further indicating that the more stem-like cells populating the core are the source of the bioluminescence.

**Table 1:** Cell types identified by markers for stem cells and differentiated cells after 1 or 4 days in serum medium.

Cell type	Day 1	Day 4
SOX2^+^	74.83 ±14.93% (n = 6)	42.00 ±6.40% (n = 7)
Msi1^+^	81.21 ±14.90% (n = 5)	50.11 ±12.77% (n = 7)
Nestin^+^/ GFAP^+^	N.A.	47.35 ±10.09% (n = 6)
Beta-III^+^	N.A.	0.62 ±1.32% (n = 7)
NeuN^+^	N.A.	0.16 ±0.27% (n = 9)

DG neurospheres were allowed to differentiate in SM for 1 or 4 days as indicated. Shown are the average percentages of cells positive for cell markers followed by standard deviation. Total numbers of spheres analyzed are indicated in parentheses. N.A.: not available.

### Circadian clock proteins are required for normal neurosphere formation

Critical genes such as *Bmal1* (*Arntl*) serving in the timing mechanism of the circadian clock are expressed in the SGZ, but studies of knockout mice lacking *Bmal1* indicate that circadian rhythms are not required for successful embryonic or adult neurogenesis [1]. *Bmal1* ^*-/-*^ knockout mice show arrhythmic locomotor activity under free-running conditions such as constant darkness (DD) [2, 33]. To determine whether a functioning circadian clock is necessary for neurosphere formation, we prepared spheres from both DG and SVZ of WT and *Bmal1* ^*-/-*^ mice.

**Fig 3:**
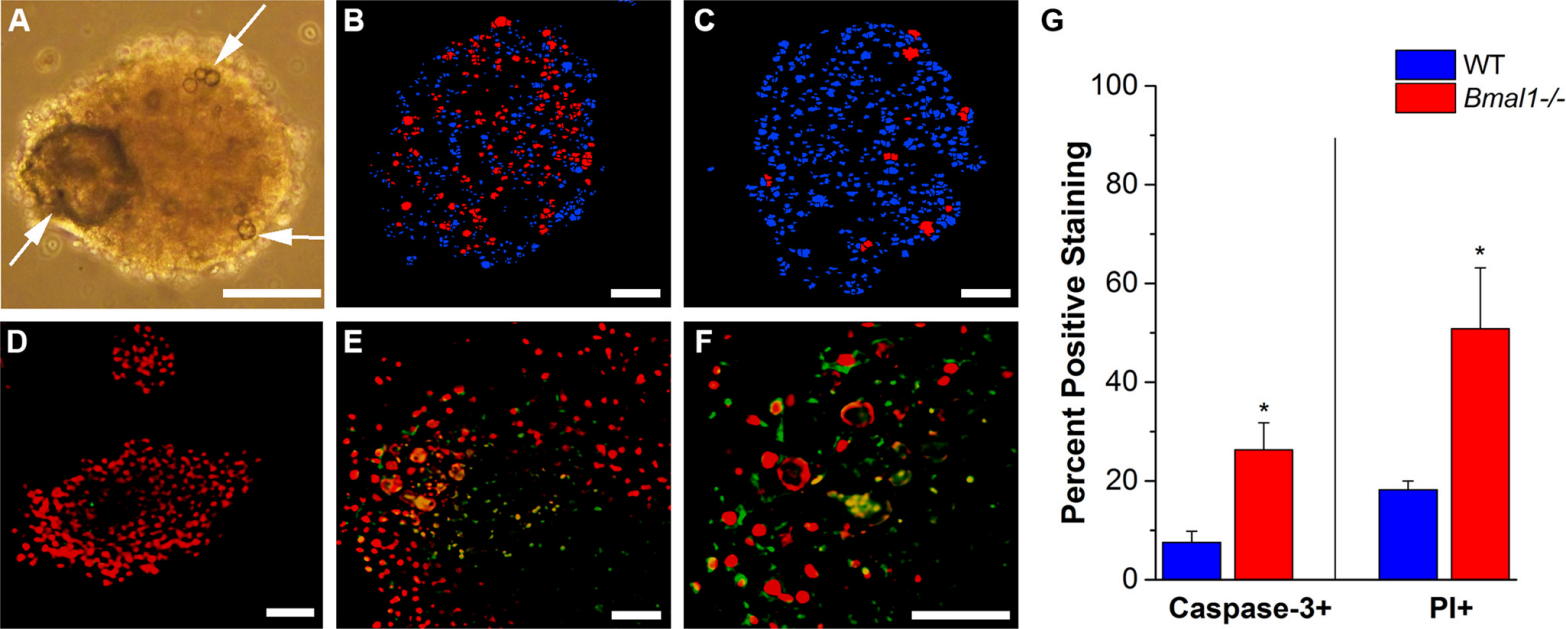
*Bmal1* ^*-/-*^ neurospheres show altered growth patterns and increased cell death. **A:** brightfield image of *Bmal1* ^*-/-*^ DG neurosphere (arrows indicate large and small lacunae). Live/dead stain using propidium iodide (red) and Hoechst3342 (blue) shows higher cell death near the lacunae in **B:** *Bmal1* ^*-/-*^ DG neurospheres when compared to **C:** WT controls. After 2 days of differentiation in SM, caspase–3^+^ cells were detected in **D:** WT neurospheres and *Bmal1* ^*-/-*^ neurospheres at **E:** 20x and at **F:** 40x magnification. Nuclei were stained with propidium iodide in (**D-F**). **G:** Percentage of caspase3+ and propidium iodide-positive cell staining in DG neurospheres from *Bmal1* ^*-/-*^ and WT littermates. Scale bar = 50 μm.

Neurospheres could be cultured from both WT and *Bmal1* ^*-/-*^ knockout animals, but distinct differences were observed in neurosphere morphology. An unusual feature was the presence of large oval structures that appear in DG neurospheres from *Bmal1* ^*-/-*^ mice after they are maintained in SCM for 15–20 days with medium exchanges every 2–3 days. These unusual structures were dark when observed in brightfield at low magnification and were referred to as “lacunae” because they were devoid of cells (Fig 3A). The average percent area occupied by lacunae was 5.15 ±4.89% (n = 26) when entire spheres were examined. DG and SVZ neurospheres from WT mice and SVZ neurospheres from *Bmal1* ^*-/-*^ mice lacked any evidence of lacunae.

Live neurospheres were stained with PI to reveal dead or dying cells and were then fixed and stained with Hoechst3342 to identify all cell nuclei. Rather than distinct cells, only diffuse fluorescence was observed in the lacunae. We quantified the total number of live and dead cells using a multi-wavelength cell scoring routine (Fig 3B, 3C and 3G). The percentage of dead cells in *Bmal1* ^*-/-*^ neurospheres increased significantly relative to WT (KO: 50.86 ±24.6%, WT: 18.2 ±3.61%; t = 2.62, p = 0.03, n = 4 spheres each). There was no obvious difference in neurosphere size (as largest cross-sectional area) between *Bmal1* ^*-/-*^ and WT neurospheres when made from either DG or SVZ.

### Absence of circadian clock proteins results in greater cell death

Because of the aberrant morphological features (lacunae) observed in DG neurospheres, we evaluated whether the circadian clock plays a role in regulation of cell death by measuring cleaved caspase–3 staining intensity and the percentage of caspase–3^+^ cells in WT (Fig 3D) and *Bmal1* ^*-/-*^ (Fig 3E and 3F) neurospheres. Neurospheres were allowed to differentiate on poly-D-lysine-coated dishes in SM for 1 day before they were fixed for immunocytochemistry. There was a significant increase in caspase–3 overall mean staining intensity in *Bmal1* ^*-/-*^ neurospheres (442.78 ±517.85 relative light units, n = 9) as compared to WT neurospheres (71 ±83.97, n = 9, t = 2.12, p = 0.049). We also recorded a significant increase in the total percentage of cells positive for cleaved caspase–3 (Fig 3G) in *Bmal1* ^*-/-*^ neurospheres (KO: 26.30 ±17.35%, n = 10; WT: 7.60 ±6.25%, n = 8, t = -2.88, p = 0.01).

### Circadian clock proteins are required for normal neurosphere growth and proliferation

**Fig 4:**
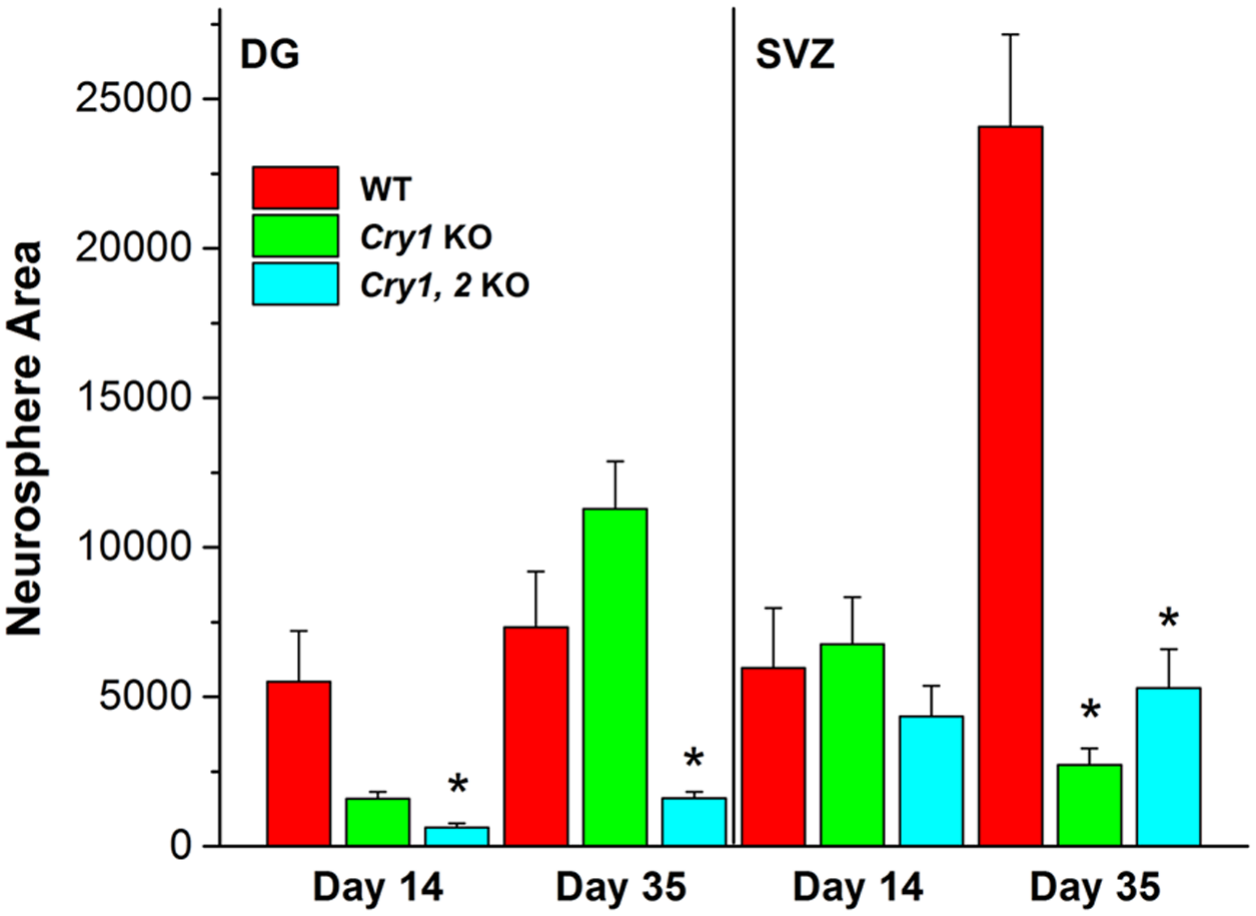
*Cry1* ^*-/-*^, *2* ^*-/-*^ neurospheres have reduced proliferation and growth in culture. The average size of DG and SVZ neurospheres from *Cry1* ^*-/-*^, *2* ^*-/-*^, WT, and *Cry1* ^*-/-*^ mice were compared at days 14 and 35 in vitro while in SCM. Shown is the total area of all spheres of each dish (as μm^2^). Asterisk indicates significant difference from the respective control (p<0.05).

In order to test whether observed defects in the neurosphere homeostasis were caused by clock disruption or by some clock-independent function of BMAL1, we decided to test properties of neurospheres generated from the DG and SVZ of another circadian mutant. We used *Cry 1* ^*-/-*^, *2* ^*-/-*^ mice that are arrhythmic in DD but maintain daily activity patterns while in 24-hr light/dark cycles [34, 38]. To determine whether a functioning circadian clock is necessary for overall growth and proliferation of neurospheres, we counted and measured cross-sectional area of spheres from *Cry 1* ^*-/-*^, *2* ^*-/-*^, *Cry1* ^*-/-*,^ and WT mice. In the absence of both circadian clock CRY proteins we found significantly reduced cell proliferation and lower numbers of secondary neurospheres (Fig 4). Secondary DG spheres from *Cry 1* ^*-/-*^, *2* ^*-/-*^ mice were on average smaller but not significantly different from the *Cry1* ^*-/-*^ controls after 14 days in culture but were significantly different at the 35^th^ day in culture (F_2,62_ = 7.91, p<0.01). No significant difference was recorded in SVZ neurosphere cultures.

To determine whether the observed effects on proliferation were in response to absence of one clock protein (*Cry1* ^*-/-*^) or a non-functional circadian clock (*Cry 1* ^*-/-*^, *2* ^*-/-*^) we also tested neurosphere proliferation in WT age-matched littermate controls. Slower neurosphere growth and proliferation were observed in *Cry 1* ^*-/-*^, *2* ^*-/-*^ mice as shown by significantly smaller DG neurospheres from *Cry 1* ^*-/-*^, *2* ^*-/-*^ mice compared to WT littermates (Fig 4) at Day 14 (F_2,28_ = 5.54, p<0.001) and at Day 35 (F_2,62_ = 7.91, p<0.01). On the other hand, no significant difference was observed in SVZ neurosphere proliferation at day 14, but proliferation was significantly reduced in both the *Cry 1* ^*-/-*^, and *Cry 1* ^*-/-*^, *2* ^*-/-*^ knockout SVZ spheres at Day 35 in culture (F_2,59_ = 44.41, p<0.001). No lacunae were observed in neurospheres made from any of the *Cry* knockout mice.

### BMAL1 is essential for neuronal fate commitment

**Fig 5:**
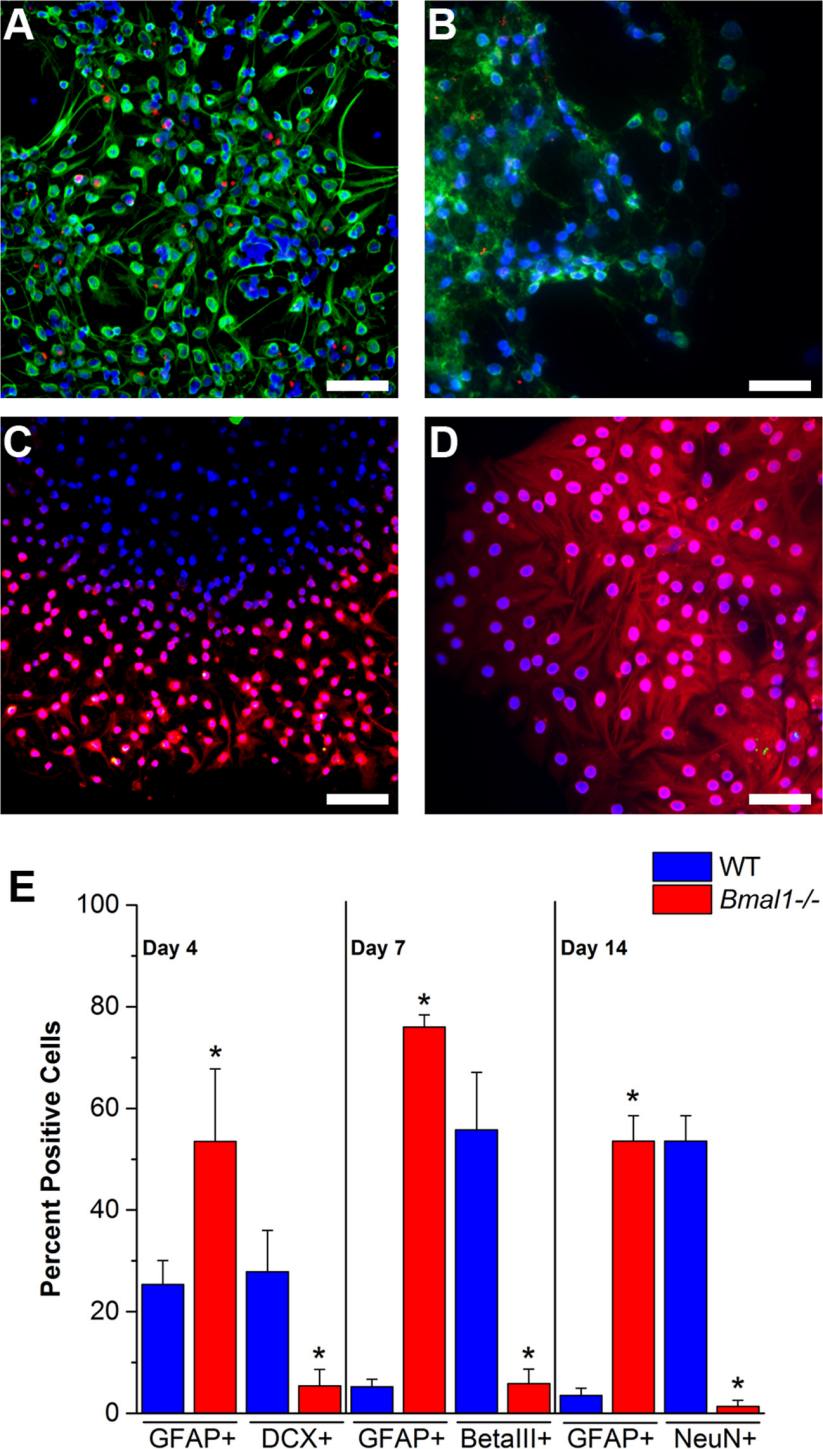
Neuronal commitment is diminished in *Bmal1* ^*-/-*^ DG neurospheres. In WT DG neurospheres the sequence of cell types during differentiation in B27 medium parallels events during in situ neurogenesis. **A:** Immature neurons expressing BetaIII-tubulin (green) at day 7 and lacking GFAP co-localization (red). **B:** Mature neurons expressing NeuN (green) at day 14 and lacking GFAP (red). In contrast, *Bmal1* ^*-/-*^ neurospheres displayed reduced neuronal differentiation and increased astrocyte proliferation. **C:** Lack of BetaIII-tubulin expression (green) shown with GFAP (red) in a *Bmal1* ^*-/-*^ neurosphere at day 7. **D:** Lack of NeuN (green) shown with GFAP (red) at day 14. All nuclei were stained with Hoechst (blue). Scale bar = 50 μm. **E:** Percentage of positive cells for DCX (neuroblasts), BetaIII-tubulin, and NeuN at days 4, 7, and 14 after differentiation in B27 medium, respectively.

To analyze whether *Bmal1*, an essential component of the circadian clock, is necessary for neurogenesis in vitro, both secondary DG neurospheres from WT and KO dishes were transferred to new 35mm poly-D-lysine-coated Mattek dishes with neural differentiation medium (B27M). Neurospheres were allowed to differentiate for 4 or 6–7 days in B27M with no added stem cell-maintaining growth factors (bFGF and EGF). Immunocytochemistry was used to determine the percentage of neuroblasts (DCX^+^), immature neurons (BetaIII-tubulin^+^), and astrocytes (GFAP^+^) in the culture. The percentage of DCX^+^ cells in *Bmal1* ^*-/-*^ DG spheres was significantly lower relative to WT controls after 4 days of differentiation in B27M (KO: 5.42 ±7.91%, n = 6; WT: 27.86 ±21.53%, n = 7; t = 3.42, p = 0.01). When compared with WT (Fig 5A) the percentage of immature BetaIII^+^ neuronal cells, after differentiation in B27 medium at day 7, was significantly reduced in *Bmal1* ^*-/-*^ DG spheres (Fig 5C) (KO: 5.88 ±8.56%, n = 9; WT: 55.79 ±7.38%, n = 8; t = 4.93, p = 0.001). In addition, these differentiated neurospheres exhibited an increased astrocyte proliferation when compared to their WT littermates (KO: 76.01 ±7.09%, n = 9; WT: 5.22 ±4.19%, n = 8; t = 32.13, p<0.001).

To rule out the possibility of delayed neuronal differentiation in the knockout cultures that would not have been detected and to further determine whether BMAL1 regulates terminal differentiation of neuronal cells, we allowed differentiation of the neurospheres in B27M for up to 14 days. Confocal immunocytochemistry was performed using anti-NeuN antibody to calculate the percentage of mature neuronal cells in the culture. We observed an obvious decline in the number of fully mature neuronal cells in differentiated *Bmal1* ^*-/-*^ DG neurospheres relative to WT (Fig 5B and 5D). To test whether loss of circadian timing could explain these results we also examined *Cry 1* ^*-/-*^, *2* ^*-/-*^ neurospheres given B27M for 14 days. When comparing both of the arrhythmic knockouts with 6-month-old and 12-month-old WT cultures (Table 2) there were significantly fewer NeuN^+^ cells in *Bmal1* ^*-/-*^ but not *Cry 1* ^*-/-*^, *2* ^*-/-*^ (F_3,34_ = 5.544, p = 0.003) neurospheres. Nevertheless, both *Bmal1* ^*-/-*^ and *Cry 1* ^*-/-*^, *2* ^*-/-*^ had significantly more GFAP^+^ cells than WT (F_3,34_ = 4.82, p = 0.006). These results, displayed in Fig 5E, indicate that neuronal fate commitment depends on non-clock functions of BMAL1, whereas glial proliferation is regulated by a circadian-dependent process, because it was observed in both knockouts.

**Table 2:** Percent positive mature neuronal and glial cells after 14 days of differentiation in B27 medium.

Genotype	Percent NeuN^+^ cells	Percent GFAP^+^ cells
*Bmal1* ^*-/-*^	1.39 ±3.44% (n = 10)	53.58 ±15.85% (n = 10)
*Cry1* ^*-/-*^, *2* ^*-/-*^	41.04 ±9.07% (n = 12)	23.41 ±8.98% (n = 12)
C57 BL6 (6-month-old WT)	53.56 ±15.84% (n = 8)	3.55 ±4.00% (n = 8)
*mPer1*::*luc* (12-month-old WT)	49.75 ±7.13% (n = 9)	9.79 ±10.18% (n = 9)

Neurospheres were made from the DG tissue harvested from mice and were allowed to differentiate in B27 medium for up to 14 days. Shown are the percentages of positive mature neuronal (NeuN^+^) and astrocyte (GFAP^+^) cells followed by standard deviation. N: the total number of spheres analyzed.

To test for the possibility that the paucity of mature neurons in *Bmal1* ^*-/-*^ cultures was caused by previously described accelerated aging due to this mutation [39] neurospheres were prepared from 12-month-old WT mice. Both groups of neurospheres were maintained in B27M for 14 days and examined for NeuN^+^ and GFAP^+^ cells (Table 2). Only the *Bmal1* ^*-/-*^neurospheres displayed significantly reduced evidence of neuron formation.

## Discussion

### Circadian clocks in dentate gyrus neurospheres

Bioluminescence imaging revealed circadian *mPer1* activity in neurospheres maintained in serum medium. In contrast, spheres in SCM were arrhythmic. Furthermore, serum medium and B27M produced the temporal sequence of stem cell markers, neuron-specific proteins, and morphological changes predicted from previous studies of DG neurospheres undergoing differentiation and development in vitro. These results point to a strong dependency of circadian timing on the release of DG stem cells from molecular processes that maintain the stem cell state.

The lack of neurosphere rhythms in the undifferentiated state resembles the failure of embryonic stem cells to show circadian rhythms in gene expression until they are induced to differentiate [31]. Although it is possible that a small number of cells within SCM neurospheres were rhythmic but not detected, functioning of the circadian clock was suppressed overall perhaps by genes that maintain cell stemness, as suggested for SVZ neurospheres [35]. Alternatively, growth factors in SCM might inhibit the clock mechanism by excessively stimulating signal transduction pathways that are used in entraining the circadian clock to external 24-hr cycles, as proposed in a study of cancer stem cell tumorspheres [40]. These glioma tumorspheres nevertheless remained rhythmic in SCM, whereas circadian rhythms were absent in DG neurospheres indicating a greater suppression of timing processes.

Circadian rhythms were evident while DG neurospheres were differentiating in SM, as shown by immunocytochemistry identifying the stem cell markers SOX2, Msi1, Nestin, and GFAP. For example, 42 ±6.40% of neurosphere cells were positive for SOX2 after Day 4 in SM. However, no mature neuronal cells were present while circadian rhythms were detected, suggesting that rhythms emerged in the NSPCs. This result was supported by mPER1 immunostaining indicating that bioluminescence originated in the stem cell-rich core of neurospheres. These outcomes support a view that NSPC subpopulations in neurospheres are circadian clock cells, in contrast to earlier studies asserting that circadian clocks first begin in mature cells and are not operational in immature, differentiating cells [41, 42]. However, additional support that differentiating stem cells are circadian is found in studies of hematopoietic stem cells, embryonic cells of the hypothalamic suprachiasmatic nucleus (SCN), and early embryonic stem cells that express circadian rhythms [43–45].

High-frequency oscillations in *mPer1*::*luc* expression were reported in SVZ neurospheres maintained in SCM [35] but were observed only once in the present study. Together, these two studies suggest that at least one of the core circadian clock genes can be modulated at higher frequencies much like the rhythmic expression of genes regulating early developmental events [46, 47]. An alternative possibility is that the ultradian oscillations are formed from the output of two circadian cell populations that remain fixed in a phase relationship about 12 hours apart, similar to descriptions of other ultradian oscillations [48, 49].

Circadian rhythms were present as early as the second day after transfer to SM, permitting adequate time to evaluate their properties. The average period of DG neurospheres in SM was shorter than 24 hrs, similar to that of the free-running circadian locomotor rhythm of the inbred C57BL/6 mouse line used in this study (23.84 hrs) [50]. On the other hand, hippocampal explant cultures from transgenic mice expressing a fusion protein of mPER2 and firefly luciferase display a circadian rhythm in bioluminescence of 25.08 hrs [14]. The phase of the neurosphere circadian rhythm was determined from the time of peak *mPer1* expression. In SM, this phase occurred as predicted following the forskolin treatment that was used to synchronize NSPCs [51]. The average peak bioluminescence occurred at intervals about 24 hrs after the forskolin pulse ended, indicating an ensemble rhythm from multiple oscillating cells [40].

One model supported by the DG results here and previous work on SVZ neurospheres [35] springs from NSPC heterogeneity and could explain the presence of rhythms during early differentiation events: At least two cell populations are considered, one that is non-circadian but substantial, and a second much smaller population that is circadian and entrains to the forskolin pulse. During differentiation, the minor population proliferates, producing a detectable ensemble circadian rhythm, whereas the non-circadian cells are diminished or depleted through asymmetric cell division. One possibility is that the original non-circadian cells are activated radial glial cells that are lost as they differentiate into NSPCs that entrain to the circadian cell population as proliferation proceeds. This two-cell-pool model describes emergence of circadian rhythms in *mPer1* expression during neural differentiation and merits further testing. It predicts that circadian NSPCs can entrain to each other through cell contacts or paracrine factors, a premise supported by cell interactions reported in tumorspheres [40].

Although very few studies have examined circadian rhythms in the hippocampus maintained in vitro, evidence indicates that it contains a peripheral circadian oscillator distinct from that of the SCN [14]. However, a different in vitro study of clock genes in isolated hippocampal cultures did not detect circadian rhythms [15, 52]. Most studies of hippocampal circadian rhythms have analyzed rhythms in DG tissue harvested at intervals from animals housed in standard cycles of 12 hrs light and 12 hrs dark (LD) [15, 23, 53]. In one case, mice were in DD for at least 2 days before dissection, thereby avoiding immediate effects of entraining light signals on gene expression [1]. Nevertheless, indirect effects from circadian locomotor activity or circadian oscillators elsewhere in the brain could have been responsible for much of the rhythmic hippocampal activity observed in earlier studies. Circadian rhythms in neural inputs to the hippocampus or rhythms in metabolic substrates and cortisol in the brain are a few ways by which circadian rhythms may be driven [54]. By avoiding these external influences, the DG neurosphere rhythms show that hippocampal cells are indeed capable of endogenous circadian timing. Furthermore, cell phenotypes present in rhythmic neurospheres are identifiable by immunofluorescence thereby suggesting which cells generate the BLI rhythm, such as the mPER1-positive cells localized to the core.

Fluorescence imaging of fixed hippocampal sections of transgenic mice expressing a DsRED and PER2 fusion protein are reported to show circadian rhythms in DG cells that appear to be quiescent NSPCs (Type 1 cells, Sox2^+^/GFAP^+^) [1]. Evidence that the rhythmic cells were Type 1 cells was indirect: High DsRED-PER2 fluorescence was inversely correlated with intensity of staining for Ki–67, a mitotic activity marker, suggesting that the circadian rhythm originated in quiescent cells. However, DCX^+^ cells were also identified as Ki-67-negative, providing an additional possible source of the rhythm [1]. Neuroblasts are DCX^+^ cells and undergo asymmetric cell division to become neurons but are distinct from Type 1 cells, which are multipotent and less differentiated [55]. An additional concern is that DsRED-PER2 rhythms that were recorded could have resulted from a rhythm in cell abundance in the DG, as purported, or from a circadian modulation of *mPer2* promoter activity.

### Circadian clock proteins and neurosphere growth and formation

Using *Bmal1* ^*-/-*^ and *Cry 1* ^*-/-*^, *2* ^*-/-*^ arrhythmic mice, we found that the circadian clock is required for normal neurosphere growth and differentiation. Slower growth rates were observed in secondary neurosphere cultures derived from DG of *Cry 1* ^*-/-*^, *2* ^*-/-*^ mice when compared with age-matched *Cry1* ^*-/-*^ mice or WT littermates. The *Cry1* ^*-/-*^ control mice are rhythmic but the period of their circadian locomotor rhythm is 1 hour shorter than WT [34]. Neurospheres generated from *Cry 1* ^*-/-*^, *2* ^*-/-*^ mice were significantly smaller and fewer relative to controls, indicating that the lack of circadian timing production or absence of other functions for cryptochrome proteins can suppress growth rates or induce increased apoptosis.

Deficits were also observed in *Bmal1* ^*-/-*^ DG neurospheres after culture in SCM for 15–20 days: Lacunae were abundant in *Bmal1* ^*-/-*^ DG neurospheres but were not present in DG neurospheres from WT littermates. Similarly, *Bmal1* ^*-/-*^ SVZ neurospheres lacked lacunae, suggesting that DG NSPCs may be more sensitive than SVZ spheres to metabolic stress or other challenges to survival in culture imposed by a loss of circadian timing. When compared with WT controls, *Bmal1* ^*-/-*^ DG neurospheres had increased numbers of PI-positive cells, indicative of damaged cells, particularly near the lacunae.

Increased levels of ROS have been reported in *Bmal1* ^*-/-*^ mice [56]. It is possible that *Bmal1* ^*-/-*^ DG neurospheres also generate more ROS than WT spheres or are more sensitive to ROS stress, leading to apoptosis. These possibilities are supported by the higher overall immunofluorescence intensity for the apoptotic marker caspase–3 that we observed in *Bmal1* ^*-/-*^ KO spheres. The percentage of cells positive for caspase–3 was also significantly elevated relative to the control at the coverslip-level of confocal imaging sections, beneath attached neurospheres. Access to culture medium would be low at this location, again suggesting that loss of *Bmal1* leads to greater sensitivity to stressors and increased cell death when cell survival is challenged [39, 57].

A previous study of the circadian oscillator in mouse embryonic fibroblasts found that loss of *Cry1*, *2* caused increased cell proliferation, but only under hypoxic conditions [58]. On the other hand, there were no changes in cell proliferation of cryptochrome knockout cells in normal culture conditions [59]. Surprisingly, we observed reduced neurosphere expansion, a possible indicator of cell proliferation, in *Cry 1* ^*-/-*^, *2* ^*-/-*^ neurospheres, which might reflect differences between cell cultures and neurosphere culture conditions or between fibroblasts and NSPCs. Nevertheless, studies should examine cell proliferation specifically in the core of *Cry 1* ^*-/-*^, *2* ^*-/-*^ neurospheres because this area is typically more hypoxic.

There is a dearth of information about *Bmal1* ^*-/-*^ cells in culture. Recent studies using *Bmal1-* modified cells suggest that the circadian clock alters the mitotic rate in different ways depending on cell type: Hepatocytes cultured from *Bmal1* ^*-/-*^ animals exhibit a delay in the G1-S phase of the cell cycle [4], whereas overexpression of this protein in NIH3T3 cells increases the cell proliferation rate [29]. One study showed an increase in proliferation in the subgranular zone of *Bmal1* ^*-/-*^ animals [1], whereas another reported normal proliferation and enhanced cell survival in the SGZ in vivo [2]. We did not observe an increased proliferation rate in *Bmal1* ^*-/-*^ DG or SVZ neurospheres. Sphere sizes were not significantly different from those of WT animals.

### Circadian clocks and neural differentiation

Recent studies identified links between core circadian clock genes and factors that regulate adult neurogenesis. One report showed that *Rev-erbα* regulates neurogenesis through *Fabp7* modulation [60]. NeuroD1, a neurogenic transcription factor, has been shown to be regulated by the BMAL1/Clock complex. The authors also reported a decline in the percentage of cells positive for the neuronal marker MAP2 after cell transfection with BMAL1 siRNA [41]. This interesting result agrees with the current study in which *Bmal1* ^*-/-*^ neurosphere cells failed to generate neurons while in B27M, a neuronal differentiation medium. We confirmed our results using immature (BetaIII-tubulin^+^ and DCX^+^) and mature (NeuN^+^) neuronal markers and recorded very few cells of either phenotype.

In contrast, there were increased numbers of GFAP^+^ cells when *Bmal1* ^*-/-*^ neurospheres were cultured in B27M. Our results also agree with a recent study in which increased astrocyte numbers were observed in cerebral cortex and hippocampus of 6-month-old *Bmal1* ^*-/-*^ mice. Disruption of the circadian clock in the brain by deletion of *Bmal1* was also shown to induce oxidative stress, astrogliosis, degeneration of axon terminals, and loss of neurons [28].

Some of the features observed specifically in *Bmal1* ^*-/-*^ neurospheres, including lacunae, could be attributed to loss of a non-circadian function of the protein rather than loss of circadian timing. If these phenomena were caused by loss of clock-dependent processes, they should have appeared in *Bmal1* ^*-/-*^ and *Cry 1* ^*-/-*^, *2* ^*-/-*^ spheres because both lack a functioning clock, but they were only associated with *Bmal1* ^*-/-*^. Alternatively, as described below, the differences between the two knockout sphere types could have been because their circadian clocks were arrested in two different ways, consequently acting at separate phases of the circadian cycle and causing levels of the many clock-controlled proteins to also differ. Interestingly, the effect on neurosphere growth from loss of *Cry1* and *Cry2* also may depend on a non-clock function of CRY1 because, like DG and SVZ *Cry 1* ^*-/-*^, *2* ^*-/-*^ spheres, SVZ *Cry 1* ^*-/-*^ spheres were significantly smaller after 35 days of differentiation even though circadian rhythms should not have been eliminated. These results suggest different but overlapping roles for CRY1 in DG and SVZ neurospheres.

Whether it was caused by loss of timing or not, the low numbers of BetaIII-tubulin^+^, DCX^+^, and NeuN^+^ cells along with the increased numbers of GFAP^+^ cells in our cultures confirmed that *Bmal1* is essential for normal neurogenesis. Along with the previously mentioned reports examining in situ neurogenesis, we conclude that fate determination in differentiating neurospheres depends on BMAL1.

Our results, as shown in Table 2, do not agree with a study by Bouchard-Cannon et al. that reports increased cell proliferation in 40-day-old *Bmal1* ^*-/-*^ animals, based on expression of the mitotic marker Ki67 and NeuN^+^ [1]. The observed difference from our study might be due to the younger age of animals they used. Another study reported no effect on proliferation in 60-day-old *Bmal1* ^*-/-*^ animals [2]. There are many possible pathways by which the clock might regulate neurogenesis. The circadian clock could directly affect differentiation through its control of an E-box element in the promoter region of neurogenic transcription factors such as NeruoD1, Pax6, etc. [41]. The promoter region of the NeuroD1 gene contains nine E-boxes [61, 62]. Circadian clocks may also regulate fate commitment by modulating miRNAs. For example, the Clock/BMAL1 heterodimer regulates miRNA 219 [63] that promotes oligodendrocyte differentiation [64]. Our results indicate that loss of BMAL1 in NSPCs suppresses neuronal fate commitment and may direct the NSPCs toward an astroglial lineage.

### Insights from DG neurospheres

The effects on growth of neurospheres we observed in response to both circadian clock knockouts indicate that the proteins serving in the oscillator’s timing mechanism are also important in neurogenesis. If, on the other hand, effects were only found in spheres from one knockout but not the other, it would be clear that the clock is not needed for normal neurogenesis to proceed. Nevertheless, the different effects on NSPCs observed in the two types of knockout neurospheres does suggest that they may be caused by deficits unique to the missing proteins. It seems equally likely that these differences are because of the different roles played by the proteins in the timing mechanism. The proteins serve at different phases of the circadian cycle many hours apart [65], and the protein expression patterns they induce are also distinct. The phenotypes that the knockout spheres exhibit appear to be because of the missing circadian timing and the unique state of clock proteins each knockout generates. For example, *mPER2* protein levels are elevated and BMAL1 is at low levels in *Cry1* ^*-/-*^, *2* ^*-/-*^ mice [66]. How the many downstream, clock-controlled genes that are under *Bmal1* timing control [67] respond differently to suppressed rather than absent *Bmal1* might explain why neurosphere features produced by the two knockouts differ: some of these effector genes may be induced under one condition but not the other.

Neurosphere cultures are also quite informative because they display endogenous capabilities and behaviors of NSPCs that can proceed independently of the neural regulation that controls stem cell proliferation and differentiation in the brain. Similar to the developing embryonic brain, adult neurosphere cultures reveal mechanisms by which neural cells arise solely from glial-like cell origins [68]. The sequence of progenitor cell types in sphere cultures may occur at a different rate than in situ, but eventually the immediate precursors of mature neurons and glia appear. Whether fully functional neurons are produced from the neurospheres used here remains to be determined. Nevertheless, it is clear that the decreased proliferation, increased apoptosis, and altered cell fate observed in the knockout spheres cannot be attributed to control or lack of control through neural signaling. The tendency to produce cells with astrocyte-like rather than neuronal characteristics is quite similar to the excessive gliosis observed in the *Bmal1* ^*-/-*^ mouse brain [28]. This result indicates that a critical decision of progenitor cells in setting the neurogenic yield depends on either a circadian timing event intrinsic to NSPCs or merely to the presence of this key protein in these cells.

### Clock, neurogenesis, and memory formation

Increased hippocampal neurogenesis is correlated with higher cognitive performance in animals. Neurogenesis-related improvements have been reported in acquisition and retention of memory in spatial memory consolidation (Morris water maze, radial arm test), fear-conditioned memory, contextual fear memory, olfactory perceptual memory, and pattern separation [69–72]. Selective ablation of neural stem cells in transgenic animals or depletion of the stem cell pool by anti-mitotic treatments has been shown to alter place and object recognition memories [73–75]. Neurogenesis in the olfactory bulb has been shown to be critical for odor discrimination tasks, odor memory, and learning [76, 77]. Overall, adult neurogenesis is important for its adaptive significance–-for example, in predator avoidance, homing behavior, locating food, or identifying mates [77].

Similarly, the circadian timing system provides animals with an ability to anticipate predictable daily events that impact their survival or fitness. The core circadian clock proteins that serve in the timing mechanism are found throughout the hippocampus [23], and impairing their normal expression causes deficits in habituation, exploratory behavior, and learning [24]. *Cry 1* ^*-/-*^, *2* ^*-/-*^ mice exhibit impaired recognition memory, increased anxiety [78], and lack of time-place associations [22], although no deficits in working or long-term memory formation were reported. In contrast, *Bmal1* ^*-/-*^ mice show a diminished learning ability and have previously been reported to display phenotypes associated with accelerated aging [1, 39]. *Per2* ^*-/-*^ mice showed impaired trace-fear memory, suppressed long-term potentiation (LTP), and diminished CREB phosphorylation [14]. Equivalent effects were observed in *mPer1* ^*-/-*^ mice in which spatial memory, CREB activation, and LTP declined [23, 79], further suggesting that *Per* genes have additional effects on hippocampal functions, perhaps independent of their role in circadian timing.

The specialized ability of the hippocampus to replace its interneurons raises the possibility that a number of the described clock-related deficits are manifested through alterations in neurogenesis. The presence of circadian clock activity observed in this study during neurosphere differentiation encourages further examination of circadian protein influences on cell determination and proliferation. Circadian properties of the NSPCs could be exploited when modifying these cells to deliver treatments to the brain for correcting neurodegenerative diseases or brain trauma. For example, if differentiation into neurons and glia is gated by the clock, then the relative yield of the cell types might be manipulated through clock gene protein expression. Furthermore, by knowing the phase of NSPC rhythms in situ it may be possible to determine when the cells are least sensitive to deleterious effects of medications. Furthermore, delivery of cancer chemotherapies could be timed to a specific phase of the NSPC rhythm to minimize stem cell toxicity and impaired adult neurogenesis.
